# How have contemporary research studies used social media to recruit to digital self-help intervention research for young people’s mental health?: a mapping review

**DOI:** 10.1186/s12874-025-02636-9

**Published:** 2025-08-29

**Authors:** Sophie Dallison, Sara Munir, Emma Chubb, Harriet J. Padley, Hiu Man Fung, Debra M. Desrochers, Maria E. Loades

**Affiliations:** 1 Department of Psychology, University of Bath, Bath, UK; 2 School of Management, University of Bath, Bath, UK

**Keywords:** Recruitment strategy, Online, Youth mental health, Anxiety, Depression, Wellbeing, Emotional problems, Intervention studies, Adolescent mental health, Social media

## Abstract

**Introduction:**

Self-help, digital mental health interventions (DMHIs) are a scalable way to improve access to mental health support. Recruiting young people to research studies evaluating mental health interventions is vital for the development of effective interventions that can address the global needs-access gap. Given that many young people are digital natives, social media offers an opportunity for researchers to share study adverts. Yet, there is a lack of reporting on specifically how social media is being used for study recruitment into research studies, such as the content of posts and the frequency of posting. We aimed to map how studies evaluating self-help DMHIs for young people have used social media as a recruitment method.

**Methods:**

We systematically searched the PubMed and APA PsycInfo (and supplementary searches on Google Scholar and Cochrane) databases from 2019 to 2024 to identify published studies reporting outcomes of self-help DMHIs for 10–24 year-olds. Data was extracted on what platforms researchers used to recruit, the frequency of posting, what kind of account (personal/institutional), and the narrative content posted in study advertisements. Findings were synthesised narratively, including descriptive counts and content analysis on text-based data.

**Results:**

Of the 37 studies meeting inclusion criteria, 18 described using social media in recruitment. For five of these, social media was the exclusive recruitment strategy. Studies used a variety of social media platforms, most commonly Facebook and Instagram. Ten studies described when and/or how often they posted on social media, and six specified what type of social media account was used to post study adverts. Ten studies included the text narrative used on social media posts and these tended to include a catchy tagline, study information, and participant eligibility criteria.

**Conclusions:**

Social media is a potential recruitment avenue. When it is used, detailing how it is used, including what platform, what content, how often, from what kind of account, and using what functionality is important for transparent reporting and reproducibility. Co-designing social media recruitment strategies with young people with living experience of using social media currently could help to align current usage patterns in the target demographic with recruitment activities.

**Supplementary Information:**

The online version contains supplementary material available at https://doi.org/10.1186/s12874-025-02636-9.

## Background

Recruiting young people to research studies evaluating mental health interventions is vital to ensure that studies are sufficiently well-powered and that findings are generalisable to the intended end user population. This is a particular priority in the context of the global worsening of young people’s mental health [1] and the unmet need for support [2]. However, there are multiple barriers to recruiting young people to studies, including lack of awareness of available opportunities [3] and negative perceptions of research [4]. Failing to recruit enough young people to a study can affect the certainty with which conclusions can be drawn because of insufficient power, and limit the generalisability of the findings as selection bias may mean that the recruited sample is not representative of the intended end user population [5].

Social media offers a way for researchers to let young people know about studies, reaching them where they are already spending time and looking for information and support [6].Many young people are digital natives and are the highest users of digital technology [1], spending a large proportion of their time on social media. In the UK, data from the Office of National Statistics suggests that 97% of 16–24 year-olds used social networking sites in the last 3 months [7]. Furthermore, recent stastics from Ofcom (the UK Office of Communications) indicate that YouTube was the most used online platform among 3–17 year-olds (88%), followed by WhatsApp (55%), TikTok (53%), Snapchat (46%), Instagram (41%) and Facebook (34%) [8]. These different platforms have different purposes, functionalities, and age restrictions (see Table 1).

**Table 1:** Functionality, purpose and age limits for social media platforms

Social media platform	Functionality	Purposes	Minimum age to have account (years)
Facebook	Profile creation, friends, photo, video and text content, news feed, messaging, groups, events, marketplace	Content sharing, social interaction, entertainment, discovery	13
Instagram	Photo and video content, followers, likes, comments, stories, messaging	Content sharing, social interaction, discovery, influence	13
LinkedIn	Profile creation, connections, photo, video and text content, job advertisement and application, messaging	Content sharing, professional networking, interaction, discovery, job searching, career development	16
Reddit	Text, photo and video content, followers, discussions, comments, questions, upvoting/downvoting	Content sharing, discovery, discussion, social interaction, community development	13
Snapchat	Ephemeral photo content and messaging	Communication, entertainment, location sharing	13
TikTok	Short-form video content, followers, likes, reposts, comments, messaging, TikTok shop	Entertainment, social interaction, discovery, influence, shopping	13
WhatsApp	Messaging, group chats, voice/video calls	Communication	13
X (formerly Twitter)	Short text, image and video content, followers, reposting, likes, messaging	Content sharing, discovery, discussion, entertainment	13

Social media has increasingly been leveraged as a recruitment avenue by mental health researchers over the last 10 years [9]. Individual studies have found that social media may sometimes be more successful than previously used traditional recruitment methods [10, 11], such as mailing lists, flyers, and posters [12]. There is also evidence that social media may be helpful in reaching underserved and underrepresented young people, such as those from low socioeconomic backgrounds [13] and global majority young people [14].

Researchers need to know how to capitalise on social media as an avenue to recruit young people to mental health intervention studies. To our knowledge, there are two existing reviews exploring recruitment to mental health studies via social media [9, 11] but only one focusing on young people specifically [11]. The latter review explored social media recruitment to mental health studies broadly, including a mix of observational and intervention studies. It reported on the types of social media platforms used and any recommendations for social media recruitment within the included papers but did not provide more detailed insights into exactly how social media was used, such as the content and medium of posts or the frequency of posting. Additionally, the search criteria were dependent on social media being reported in the title or abstract and authors acknowledged that this may have screened out relevant studies that did recruit via social media. Given these gaps in the literature, our review aimed to give more details about how social media was used and identifies studies that used social media for recruitment through full-text screening for social media use. Choosing a narrower scope, by focusing on digital intervention studies specifically, would make this more approach feasible.

Studies on self-help DMHIs, whereby psychological therapy is delivered unguided, without the need for a therapist or human supporter, could be a valuable area to explore social media recruitment in more detail. These widely scalable interventions already capitalise on young people’s use of digital technologies and are particularly well suited for embedding into social media because they can often be accessed on demand and without requiring permission from gatekeepers or meeting thresholds for access. Social media enables messaging about self-help DMHIs to reach young people where they are, rather than waiting for them to actively seek out support, capitalising on moments when both their motivation and needs are high [15]. Therefore, understanding how researchers have used social media to recruit young people into self-help DMHI studies could provide useful insights for researchers into how to use social media to recruit to such studies as well as shed light on the real-world implementation of this type of intervention.

We conducted a mapping review of contemporary studies evaluating the feasibility, acceptability, and/or effectiveness of self-help DMHIs to identify if, where, and how they have used social media to recruit young people. We focused on contemporary studies (2019–2024) because of the developments of social media platforms in recent years, particularly the release of TikTok in 2016, which started gaining momentum in 2018. Specifically, we wanted to describe the platforms researchers used, how often they posted, from what account (personal/institutional), and the kinds of content they posted (visual/media and narrative text).

## Methods

We conducted a mapping review, a type of systematic approach to collating, describing and cataloging the available evidence and evidence gaps related to a question of interest [16]. We employed this method to synthesise descriptive information [16] on the recruitment of young people via social media to self-help DMHIs. A mapping review was appropriate for the current research question, as it allowed for a broader focus than other types of reviews on this previously unexplored research area. We pre-registered our protocol on the Open Science Framework before commencing our searches (DOI https://doi.org/10.17605/OSF.IO/2RFWX).

### Search strategy

Searches were conducted in August 2024. We primarily searched Pubmed and APA PsychNet, databases that index psychology and medical journals. Our search terms for the primary database searches were informed by a prior systematic review [17]. However, to address the limitations of previous work, we did not use terms related to social media recruitment as necessary for inclusion, as this may not be reported in the Title or Abstract. As such, we developed strings of search terms to search for the following concepts: ‘adolescents’, ‘digital self-help’, and ‘common mental health problems/wellbeing’. In line with the notation of each database, we used MeSH terms, titles and abstracts, and Boolean operators. Filters were only applied for the date of publication (see Appendix 2 for search terms). Following study screening, a secondary, supplementary search was conducted on Google Scholar and the Cochrane database using similar search terms to review the first 100 results per search.

### Study screening and selection

Search results were imported to Endnote for de-duplication and then to Covidence for study selection. We formulated our detailed inclusion criteria using an adapted version of the PICOS framework [18], employing PIS (population, intervention, study type), as ‘O’ for outcome and ‘C’ for comparator were not applicable to identifying the papers to include in this review (see Table 2 for inclusion criteria).

A total of four people were involved in screening for inclusion. These were primarily two doctoral level psychology students (SD, SM), who trained and supervised an additional two undergraduate psychology students. We conducted title and abstract screening to exclude irrelevant studies, followed by a full-text review of the remaining potentially eligible studies. Double screening by two reviewers was conducted for 50% of studies at both stages of screening (reviewers were blinded to each other’s decision). Any conflicts were resolved through discussion between reviewers and the eligibility criteria would be clarified and further training provided to the psychology undergraduate reviewers. We received input from the senior supervising author (ML) where required. As this was a scoping mapping review, where a team of multiple reviewers was used, inter-rater reliability of conflicts cannot be reported due to the various pairings of reviewers.

**Table 2:** Inclusion and exclusion criteria according to the PIS framework

Element		Inclusion criteria	Exclusion criteria
Population	Adolescents and young people	Participants are between 10–24 years old	Where the study includes participants outside the age range of 10–24, studies were excluded if they predominantly include participants < age 10 or > age 24, or where the mean age of sample > 22 or < 12.
Intervention	Digital self-help interventions aimed at anxiety/depression symptoms, emotional problems/wellbeing	Self-help intervention Intervention was digitally delivered, including web-based, text message-based, app-based, and artificial intelligence supported. Interventions including very limited contact with a clinician/human supporter were included only if the support was not part of the primary intervention (e.g. for research or enrolment/onboarding purposes only).	Intervention is not self-help. Intervention is face-to-face. Intervention is digital however is primarily facilitated by a clinician/trained human supporter.
Study type	Any intervention study published in a peer-reviewed journal since 2019.	All pre-post intervention studies reporting on primary research, including RCTs, pilot RCTs, pre-post designs, and case studies.	Not published in English. Not published in a peer-reviewed journal. Published prior to 1 st January 2019.

### Data extraction

Data was extracted from the papers that met the inclusion criteria into a customised data extraction template on Covidence. The data extraction template was piloted with one paper and amendments were made until the researchers were satisfied with the form. Either SD or SM extracted the data from all papers, with additional help from trained undergraduate psychology students, and conflicts were resolved by discussion. Data extracted included study characteristics (first author, country conducted, aim, study design, intervention description), sample characteristics (mean age, age range, education status, and total number), social media recruitment information (social media recruitment used, social media platforms used, frequency of posting, account posted from and narrative in posts). The extracted data was exported to Excel for data synthesis.

Where information of interest was not available, we contacted the corresponding author by email to request this (11 emails sent). We sent one follow-up email a week later if we did not receive a response. Eight responses were received and for the three studies where information was not forthcoming, we reported all the information of interest that was publicly available.

### Data analysis

We synthesised the findings narratively [19]. Studies were grouped by whether they had used social media in recruitment and whether social media recruitment was the only recruitment method used. Percentage and proportions were calculated for: social media use, social media as the only recruitment method, social media platforms used, and frequency of posting.

We initially pre-registered our intention to do a narrative synthesis and intended to use thematic analysis for this [20]. However, the data was not sufficiently rich and therefore we instead drew on content analysis principles [21] for the narrative/content of the social media posts from the papers that provided this information. Using an inductive approach to create meaningful units for the data [5], we open-coded the data to create sub-categories and then pooled these into categories, and counted the number of citations represented by each category. SD and SM compared codes to ensure reliability.

### Quality assessment

For this review, a formal quality appraisal of the included papers was not deemed appropriate or meaningful. Therefore, we did not conduct this as it is not necessarily required for scoping/mapping reviews as per the PRISMA extension relevant to this review type [22].

## Results

### Study selection

**Fig. 1:**
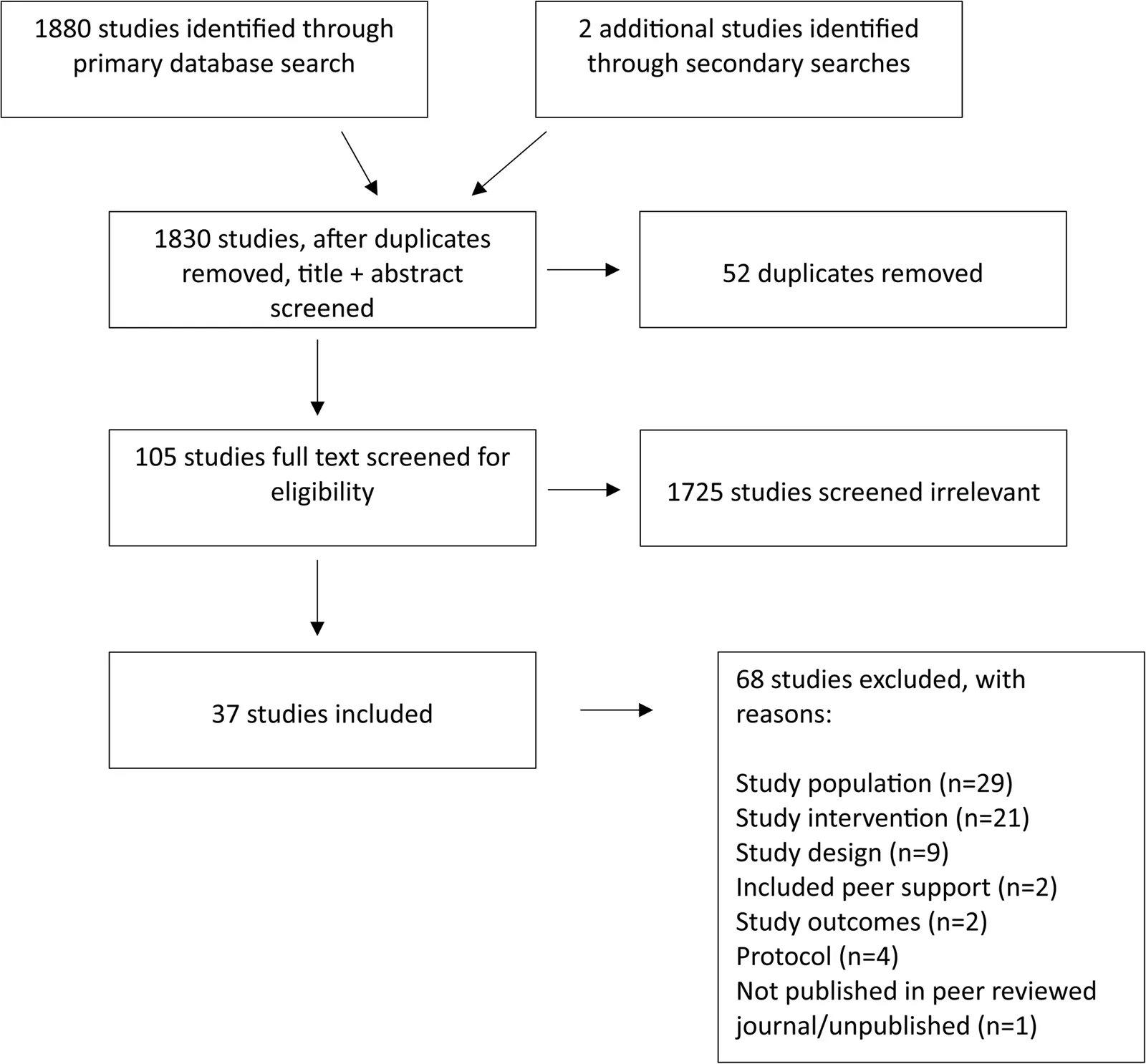
Literature search flow diagram

After the full-text review, 37 studies were included in this review (see Fig. 1): 19 RCTs, 11 feasibility/acceptability studies, 3 randomised control feasibility studies, 2 quasi-experimental designs, 1 open trial, and 1 non-randomised control.

To address to first part of our objective, to identify if studies have used social media to recruit young people, we found that 18 of the 37 studies (48.65%) mentioned the use of social media to recruit young people to the study (see Table 3). We will henceforth focus on these studies only, as the other 19 studies used alternative recruitment methods or did not detail using social media, despite contacting corresponding authors to enquire.

**Table 3:** Summary of included studies, including country, study design, sample characteristics, and intervention

Authors and year	Country	Study design	Sample: participants; age range (Mean)	Intervention
Carmona et al., 2021 [26]	Canada	Feasibility/Acceptability Study (including pre-post measures)	51; 15–24 (20.17)	DOZE: A web-based app designed to address sleep disturbance among adolescents and young adults. Included a psychoeducation, a sleep diary, personalised feedback, and goal setting.
Chang et al., 2022 [27]	United States	Randomised Controlled Trial	679; ^m^–^m^ (21.3)	A 25-minute web-based module teaching two Upa Yoga practices designed to improve student wellbeing during COVID-19. Participants were then provided with instructional videos and audio guidance and recommended to practice daily for 12 weeks.
Cook et al., 2019 [28]	England	Randomised Controlled Trial	235; 18–24 (20)	i-RFCBT: A web-based 6-module CBT intervention to address rumination by shifting thinking to become more concrete, specific and action-orientated. Included psychoeducation, mood diaries, audio exercises, and testimonials. There were two versions: guided and unguided.
Dobias et al., 2022 [29]	United States	Randomised Controlled Trial	Project ABC = 3679, Project SAVE = 1652, The REFRAME = 848; 13–19 (Project SAVE = 15.71, other conditions = ^m^)	Project ABC: A 5- to 8- minute web-based single-session intervention to boost mood and build self-efficacy by encouraging engagement in pleasurable, value-aligned behaviours. Included psychoeducation, value assessment, planning and writing exercises. Project SAVE: An 8-minute web-based single-session intervention designed to reduce self-harm behaviours. Included psychoeducation, statistics and testimonials and alternative coping strategies. The REFRAME: A 5-minute web-based single-session intervention teaching cognitive reappraisal. Included psychoeducation, testimonials, a practice exercise and prompts to apply cognitive reappraisal to their own lives.
Harith et al., 2024 [30]	Malaysia	Quasi Experimental	464; 18–25 (20.88)	EduMind: A 7-day web-based intervention tailored to participants’ individual mental health status including psychoeducation, CBT elements, breathing exercises and mood journaling. Participants were encouraged to interact with the website for 10 min daily, at least five times a week.
He et al., 2022 [31]	United States	Randomised Controlled Trial	148; 17–21 (18.78)	XiaoE: A 7-day web-based artificial intelligence chatbot-guided CBT intervention for young adults with depression. The chatbot provided daily modules on topics such as cognitive distortions, self-esteem, mindfulness, loneliness and gratitude journaling.
*Kahlon et al., 2023 [32]	Norway	Randomised controlled trial	100; 13–16 (14.2)	VRET: A self-guided 3-week virtual reality intervention for public speaking anxiety in adolescents. Involved speech exercises with virtual audience and gamified elements.
Kulikov et al., 2023 [33]	United States	Randomised Control Feasibility Trial	51; 13–21 (intervention = 17.9; control = 16.96)	Spark v2.0: A 5-week app-based BA intervention for depression in adolescents. Included psychoeducation on BA, tracking features, skill building, identifying barriers and planning features.
*Maciejewski & Smoktunowicz, 2023 [23]	Poland	Randomised Control Trial	372; 18–47 (20.98)	Stressbot: A self-guided 7-day web-based intervention aiming to reinforce coping self-efficacy, reduce stress and improve quality of life. Involved 6 daily exercises delivered as messages which users could reply to via text, voice or buttons.
*Perumbil Pathrose et al., 2022 [34]	Australia	Feasibility/Acceptability Study (including pre-post measures)	31; 14–29 (21.65)	Mindfulness-based e-book: A web-based 6-module mindfulness intervention focusing on paying attention to the five senses and self-care. The modules incorporated audios, reflection activities, and interactive quizzes.
Radomski et al., 2020 [35]	Canada	Randomised Controlled Trial	536; 13–19 (16.6)	The Breathe program: A six-session web-based CBT intervention for anxiety, which included content such as relaxation, avoidance, cognitive distortions and realistic thinking. Included risk management, psychoeducation, reflection and activities.
*Schleider et al., 2020 [36]	United States	Feasibility/Acceptability Study (including pre-post measures)	539; 11–17 (^m^)	Project Personality: A self-guided, 30-minute single-session intervention teaching how and why to adopt a growth mindset. Project CARE: A self-guided, 30-minute single-session intervention teaching how and why to increase self-compassion to reduce self-hate. The ABC Project: A self-guided, 30-minute single-session BA intervention for depression teaching how and why we should engage in valued activities.
*Serlachius et al., 2021 [37]	New Zealand	Feasibility/Acceptability Study (including pre-post measures)	41; 16–30 (21.25)	Whitu: An app-based 2-week CBT- and PP-based intervention for young people’s emotional well-being. Content included relaxation, self-compassion, gratitude and goal setting.
*Smith et al., 2023 [24]	Unites States	Feasibility/Acceptability Study (including pre-post measures)	74; 13–17 (^m^)	Project Body Neutrality; A web-based, self-guided single-session intervention that aims to increase body functionality appreciation to reduce body dissatisfaction in young people. Included psychoeducation, self-reflection exercises and writing prompts.
*Subotic-Kerry et al., 2023 [38]	Australia	Quasi Experimental	336; 12–15 (13.08)	The Bite Back Mental Fitness Challenge: A 7-module, web-based, self-guided PP intervention aiming to encourage self-reflection and personal growth. Included animations, questionnaires and activities.
*Torok et al., 2022 [25]	Australia	Randomised Controlled Trial	455; 18–25 (21.5)	Lifebuoy: a 7-module, app-based self-guided DBT intervention designed to improve emotional regulation and increase distress tolerance skills. Included psychoeducation, interactive exercises and mindfulness, breathing and self-soothing tools.
Watkins et al., 2024 [39]	United Kingdom, Germany, Spain and Belgium	Randomised Controlled Trial	2532; 16–22 (19.2)	An app-based intervention for mental wellbeing. There were two versions: one based on CBT principles and strategies, and one focused on emotional competence. They both included psychoeducation, self-monitoring, strategies, practise exercises, and gamified aspects.
Zelihic, et al., 2023 [47]	Norway	Randomised Controlled Trial	71; 11–18 (13.98)	YPF: A 7-session self-guided web-based intervention designed to increase psychosocial adjustment to having an appearance-affecting condition. Included advice, guidance and strategies for managing common challenges related to having a visible difference.

*corresponding author provided more information on social media recruitment upon request

*ABC *Action Brings Change, *AIM *Anxiety Insight Modules, *BA *behavioural activation, *CBT *cognitive behavioural therapy, *CBM-I* Cognitive Bias Modification Interpretation Retraining, *DOZE *Delivering Online Zzz’s with Empirical Support, *DBT *dialectical behavioural therapy, *i-RCBT *internet-based Rumination-focused Cognitive Behavioural Therapy, *i-TRAC *internet-based Talking About Risk and Adolescent Choices, *LIFT *Life Improvement for Teens, *MoST-MH *Mobile Support Tool for Mental Health, *OCD *obsessive compulsive disorder, *PP *positive psychology, *SAVE *Stop Adolescent Violence Everywhere, *VRET *Virtual Reality Exposure Therapy, *YPF * Young Persons Face

^m^missing data

For 3 (16.66%) of these studies, social media was the exclusive recruitment strategy [23–25] and the remaining 15 studies used other methods of recruitment in conjunction with social media recruitment [26–39]. For 8 studies, we obtained additional information on request from the corresponding author. The digital interventions used in these studies addressed a variety of mental health conditions and symptoms. Some aimed to reduce negative symptoms including sleep disturbance [26], unhealthy thinking patterns [28, 29] depression/low mood [29, 31, 33, 36], self harm [29], stress/anxiety [23, 32, 35], body dissatisfaction [24] and emotional disregulation [25]. Others focused on increasing wellbeing [27, 37, 39] through targeting factors like growth mindset [36], mindfulness [34], self-compassion [36] psychosocial adjustment [44] and self-reflection [38]. One study personalised the intervention to participants’ individual mental health statuses [30]. A full overview of studies and the interventions used is provided in Supplementary Materials Table 1.

### Social media platform

**Fig. 2:**
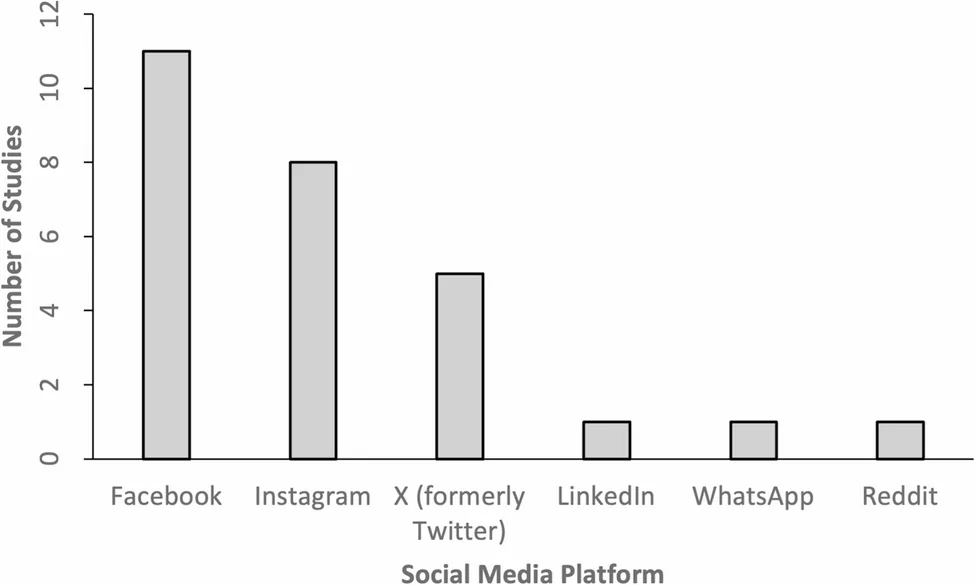
Number of studies that used each social media platform for recruitment

Six different social media platforms were used across the 18 studies. As shown in Fig. 2, the most frequently used were Facebook, Instagram, and X (formerly Twitter), while LinkedIn, WhatsApp, and Reddit were respectively used once.

### Frequency of posting advertisements on social media

Of the 18 studies, 10 included the timeframe of posting on social media [23–25, 32–38]. As shown in Table 4, the level of detail provided describing the frequency of posting varied, and the frequency of posting itself varied, with some studies recruiting over longer periods and others more intensively, and three studies explicitly mentioned using paid advertisement features within the social media platforms like paid boost posts in Instagram.

**Table 4:** Studies that included details of the frequency of posting advertisements on social media

Study ID	Frequency of posting
Kahlon 2023 [32]	Once a week or every other week
Kulikov 2023 [33]	2 months
Maciejewski 2023 [23]	Adverts were shown on Facebook for 2 months
Perumbil Pathrose 2022 [34]	August−December
Radomski 2020 [35]	Nov 2016 - July 2018
Schleider 2020 [36]	Over a couple of weeks, handful of times
Serlachius 2021 [37]	1 Paid Facebook/Instagram advert − 6–10 July 2020 (4 days) 1 post in Facebook groups
Smith 2023 [24]	Once then boosted over 3 days
Subotic-Kerry 2023 [38]	At least monthly
Torok 2022 [25]	Studies advertised in one paid for 2-week sprint (posted on social media everyday with 2 weeks).

### Social media account used to advertise (personal/institutional)

**Fig. 3:**
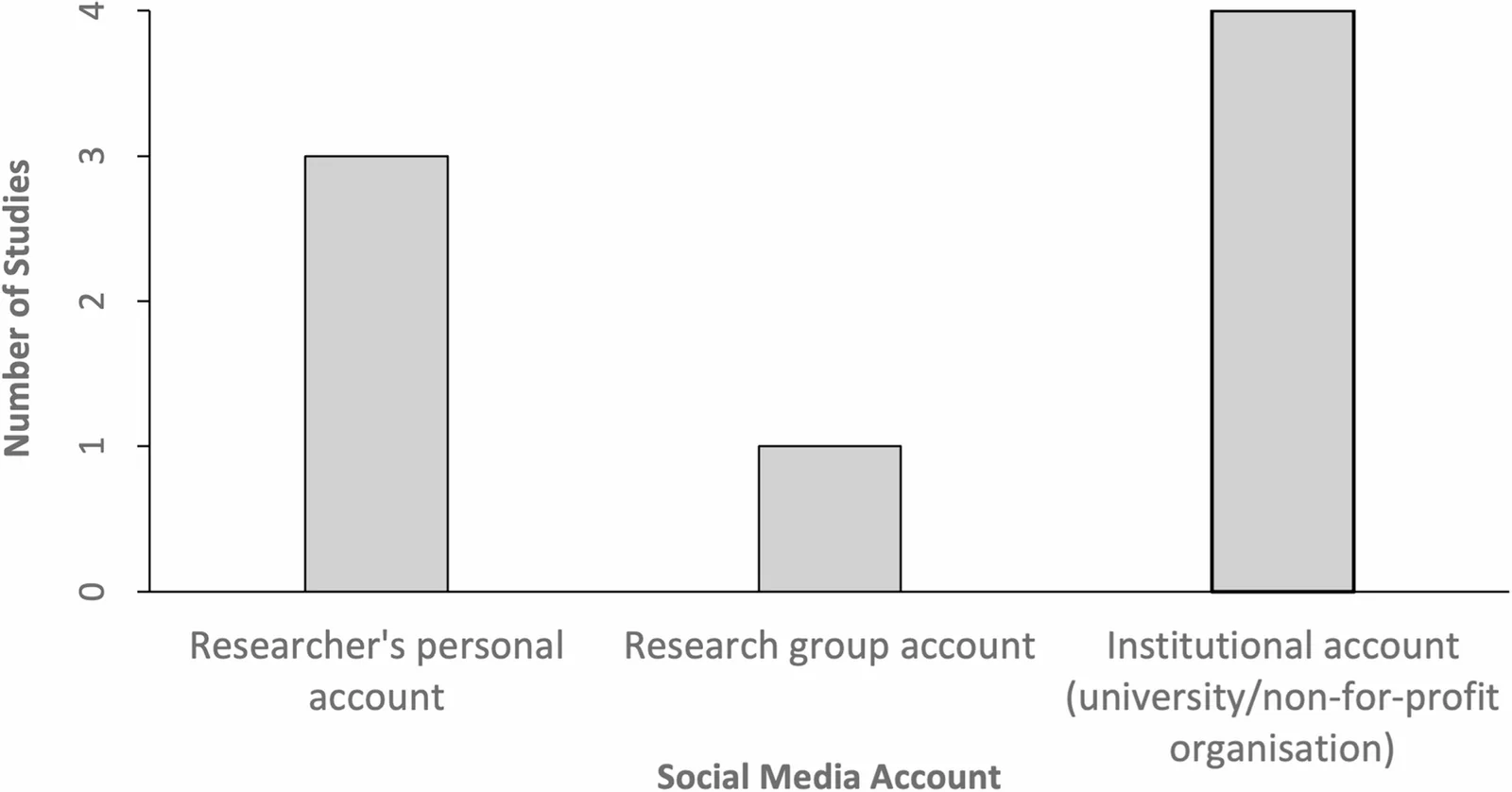
Number of studies that described which type of social media account used to advertise

Only 6 studies [11, 23, 25, 34, 37, 38] specified the type of account used to post the study adverts. Of these, institutional accounts were used most frequently (see Fig. 3).

### Content posted (visual/media and narrative text)

Ten studies [23–27, 29, 34–37] included the exact text narrative that they used on social media adverts. Table 5 summarises the categories and subcategories we derived from both the content written on the image post on social media platforms and the content from the caption alongside the post of the included studies.

**Table 5:** Content analysis categories and subcategories of studies including content posted on social media posts

Category	Sub-Category	Studies that included this
Catchy tagline	Participant wellbeing benefits	Smith [24]; Schleider [36]
	Helping others by taking part	Schleider
	Financial incentive	Perumbil Pathrose [34]
	“Are you…?”	Perumbil Pathrose; Maciejewski [23]
	Rhyming tagline	Serlachius [37]
Study information	Study/intervention aim	Torok [25]; Smith; Serlachius; Schleider; Perumbil Pathrose
	Project/study/intervention name	Smith; Serlachius; Maciejewski; Dobias [29]
	Remuneration (compensation/free)	Torok; Perumbil Pathrose; Chang [27]
	Time commitment	Torok; Smith; Carmona [26]
	What is involved for participants/nature of study	Torok; Smith; Serlachius; Perumbil Pathrose; Maciejewski; Chang
	Link/QR code to participate/more study information	Schleider; Radomski [35]; Maciejewski; Perumbil Pathrose; Torok; Serlachius
Participant eligibility	Age range	Torok; Smith; Serlachius; Perumbil Pathrose
	Target population	Perumbil Pathrose; Maciejewski
	Mental health/wellbeing symptoms targeted	Perumbil Pathrose; Carmona

### Catchy tagline

Five studies [23, 24, 34, 36, 37] that posted social media adverts included a catchy tagline in the post, which could be included to grab the user’s attention. The language used in these taglines was often short, snappy, and engaging. Two studies included the wellbeing benefits to the potential participant in the tagline, e.g. “It’s time to enjoy life without worrying about our appearances… designed to boost mood and self-image” [24] and “Teens learn new ways of dealing with stress” [36]. Schleider also used the potential of helping others in their advert tagline, “help others to do the same” [36]. Financial incentive was used in one study, “ Want to receive $60 in gift vouchers for taking part in a research study?” [34]. Two studies phrased their taglines starting with “Are you…?” questions, e.g. “Are you interested in becoming part of a research study in mindfulness? Are you aged between the ages of 14–29?” [34] and “Are you a student?” [23]. Finally, one study used rhyming in their tagline, “ 7 ways in 7 days” [37].

### Study information

All 7 studies included study information on their social media adverts, however the content and narrative of the study information varied between adverts. Five study adverts [24, 25, 34, 36, 37] included the study or intervention aim, e.g. “participate in a study evaluating the effectiveness of a smartphone app designed to reduce suicidal thoughts”. Four study adverts [23, 24, 29, 37] included the study or intervention name. Three study adverts [25, 27, 34] included details of whether participants would be compensated financially for their time or not, i.e. whether the programme was renumerated or free, as well as three including the time commitment required for the advertised study. Six study adverts described what would be involved for potential participants if they were to participate in the study [23–25, 27, 34, 37]; the detail and specifics varied between adverts, e.g. “Being involved: learn some simple mindfulness techniques, practice some skills which may help during a cancer journey” [34] and “Your involvement would include using the app for at least one week and filling out some only questionnaires about your mood, stress levels and wellbeing” [37]. Signposting to further study information and to the study website via QR code or a link to sign up was included in six study adverts [23, 25, 34–37], e.g. “click here to get more information” [25]. “Please click on the appropriate QR code above” [34]; Schleider’s [36] and Radomski’s [35] posts directed potential participants to a trial website to view more information.

### Participant eligibility

Six studies [23–26, 34, 37] included some information about participant eligibility on the social media recruitment advert. Four studies [24, 25, 34, 37] included the age range and two studies [23, 34] specified a target population, e.g. young person, health professional or parent/carer [34] and students [23]. Two study adverts [26, 34] specified mental health/wellbeing symptoms that were eligibility criteria, e.g. “young people… who have experienced suicidal thoughts” [34] and “adolescents and young adults who were dissatisfied with their sleep or were experiencing difficulty sleeping” [26]. Although not detailed in the advert itself, one study [28] stated that “Twitter and Facebook were used to circulate the advertisement to young people who expressed an interest in the following terms: stress, worry, rumination, mental health, self-esteem, well-being”.

## Discussion

Our review of contemporary self-help DMHI studies found that around half of the 37 included studies reported using social media for participant recruitment, with five studies exclusively recruiting via social media. However, there was considerable variety in the amount of detail studies contained about how social media was used. The most commonly used platforms were Facebook and Instagram, and there was considerable variety in how frequently adverts were posted, with some studies using paid adverts or boosting features within social media platforms to reach specific target audiences. Most social media adverts included key information like the study aim, who can take part, what is involved for participants, and an easily accessible link or QR code to enable interested potential participants to find out more and sign up.

To leverage the capacity of social media to reach young people where they are [40], researchers need to be cognisant of current social media usage patterns by their target population, and ensure that the platforms they are using match current usage trends. However, we note that there is some degree of a mismatch, with several of our included studies using Facebook even though data on usage patterns amongst young people indicates that TikTok, Snapchat, Instagram, and YouTube are more commonly used [8]. This may not be surprising, as the audience profile of social media platforms varies greatly across age, gender, and ethnicity [41], and researchers are likely of a different demographic (age group) to potential participants and therefore are familiar with and are users of different social media platforms. Co-designing social media recruitment strategies with young people who have living experience (i.e. are currently) of using social media as a person within the target participant demographic could help researchers to better align their recruitment strategies with the platforms young people use. In turn, this could improve the reach of study adverts and, ultimately, the degree of confidence in the findings as studies are more likely to recruit a sufficiently powered sample size, and the generalisability of the findings as the sample would be more representative of the intended end user population.

Most published papers included in our review provided limited information on their social media recruitment strategy and we had to reach out to corresponding authors for more information, with eight responses received. Although it is possible that there was more detail given about planned social media recruitment in study protocols/pre-registrations, we did not include these because we were interested in what studies actually did, rather than what they planned to do. Given how rapidly social media platforms develop and change between study pre-registration and closing recruitment, we deemed pre-registered plans to be a relatively unreliable source of information to answer our questions of interest. Given the growing body of literature highlighting that social media can be a successful recruitment strategy of young people to mental health research [11, 12], we recommend that future studies include more details of their social media recruitment strategy. Describing the social media platforms used, the frequency of posting, and the kind of account posting from (i.e. researchers’ own or organisational account) would improve repeatability. Including example materials used in social media posts in supplementary materials could further improve transparency. It would also be helpful for researchers to include reflections on the effectiveness of recruiting via social media compared to other recruitment avenues (where multiple are used). Cost-benefit analyses should be conducted [42].

Recruiting young people to mental health studies via social media presents complex ethical challenges that extend beyond traditional research frameworks. Researchers must ensure truly informed consent from participants who may have limited capacity to fully grasp the implications of research participation due to developmental stage [43]. Moreover, the blurred boundaries between therapeutic and research contexts online may lead young people to conflate participation with treatment, further complicating the ethical landscape. The digital nature of recruitment raises significant concerns around data privacy [44] and the potential for coercive or manipulative targeting through platform algorithms [45]. Whether social media recruitment is appropriate, and the specific ethical issues it raises, in part context specific, and therefore should be considered for each individual study and target population [45].

Although social media can be a means of reaching potential participants, it still may be difficult to access underserved groups, such asthose from low socioeconomic backgrounds [13] and Global Majority young people [14], thereby perpetuating existing biases in sampling. This may limit the generalisability of the findings due to the non-representativeness of the sample. This could be mitigated to some extent by having social media advertisements shared by those with enough followers to reach the intended cohorts. Given that most accounts used by studies included in our review had relatively small followings, it may be that partnering with influencers with a large reach could be a potential way forward [46]. Most included studies in our review were conducted in High Income Country contexts, so little is known about whether and how social media could be used in recruitment to mental health studies beyond these settings.

## Strengths and limitations

We systematically searched for studies that met our pre-specified inclusion criteria and used strings of search terms that were developed with input from several experts in the field, thus maximising our chances of identifying studies that met our inclusion criteria from our database searchers. Although we only used two databases for our main searches, so we may have missed potentially eligible papers that were indexed elsewhere, we did conduct supplementary searches on Google Scholar and the Cochrane database to identify relevant studies we may have otherwise missed. Although 50% of study screening and selection decisions were independently made by two review team members at both title/abstract and full-text stages, we did not formally record the number of conflicts at each stage and therefore were not able to report inter-rater reliability. Finally, we did not conduct a quality assessment of included studies, as a mapping review is to map the studies published on this topic and not relevant to our research question. However, this meant that we were unable to differentiate between higher- and lower-quality evidence and contextualise findings accordingly.

## Recommendations for future research

- Young people are on social media– meet them where they are at, recruit on the network sites that they are using– Instagram, TikTok and Snapchat.
- Researchers who are using social media for recruitment should detail what and how they use social media and calculate cost-effectiveness.
- Specific demographic groups of young people being recruited by social media to research were not provided by any study included. This is important so social media recruitment materials can be tailored to specific populations. Researchers should be mindful that some groups may be more or less likely to be reached by social media and influencers sharing information may introduce biases.
- A comparison between different types of social media adverts used is needed in order to determine whether different narrative, wording, and language on posts influences uptake.
- It is challenging to stay abreast of the rapidly evolving social media and technology field whilst research takes years to plan, get approvals for, conduct and report. The impact of TikTok in very recent years may not be reflected yet in the literature, so future research should look at how/if TikTok is being used as a recruitment tool.

## Conclusions

Our review of contemporary self-help DMHI studies found that while social media is used for participant recruitment, there is considerable variability in the detail provided about how it is used, which limits repeatability. To capitalise on the reach of social media, researchers need to align social media recruitment activities with current usage trends and preferences of their target population. To improve recruitment strategies and reach, researchers should co-design social media approaches with young people and for transparency, they should provide detailed descriptions of their methods and report the effectiveness of these, including cost-benefit analyses.

## Electronic supplementary material

Below is the link to the electronic supplementary material.

Supplementary Material 1

## Data Availability

No datasets were generated or analysed during the current study.

## Abbreviations

DMHI: Digital Mental Health Intervention
RCT: Randomised Controlled Trial
UK: United Kingdom
APA: American Psychological Association
PICOS: Population, Intervention, Comparator, Outcome, Study design
PIS: Population, Intervention, Study type
MeSH: Medical Subject Headings
OSF: Open Science Framework
