# Correction: How have contemporary research studies used social media to recruit to digital self-help intervention research for young people’s mental health?: a mapping review

**DOI:** 10.1186/s12874-026-03024-7

**Published:** 2026-10-24

**Authors:** Sophie Dallison, Sara Munir, Emma Chubb, Harriet J. Padley, Hiu Man Fung, Debra M. Desrochers, Maria E. Loades

**Affiliations:** 1 Department of Psychology, University of Bath, Bath, UK; 2 School of Management, University of Bath, Bath, UK

**Correction: BMC Med Res Methodol.** **2025;25:203**

https://doi.org/10.1186/s12874-025-02636-9

Following publication of the original article, the authors identified an error in Fig. The PRISMA figure has incorrect selection counts.

The incorrect and the correct figure is given below.

The incorrect Fig. 1 was.

**Figure:**
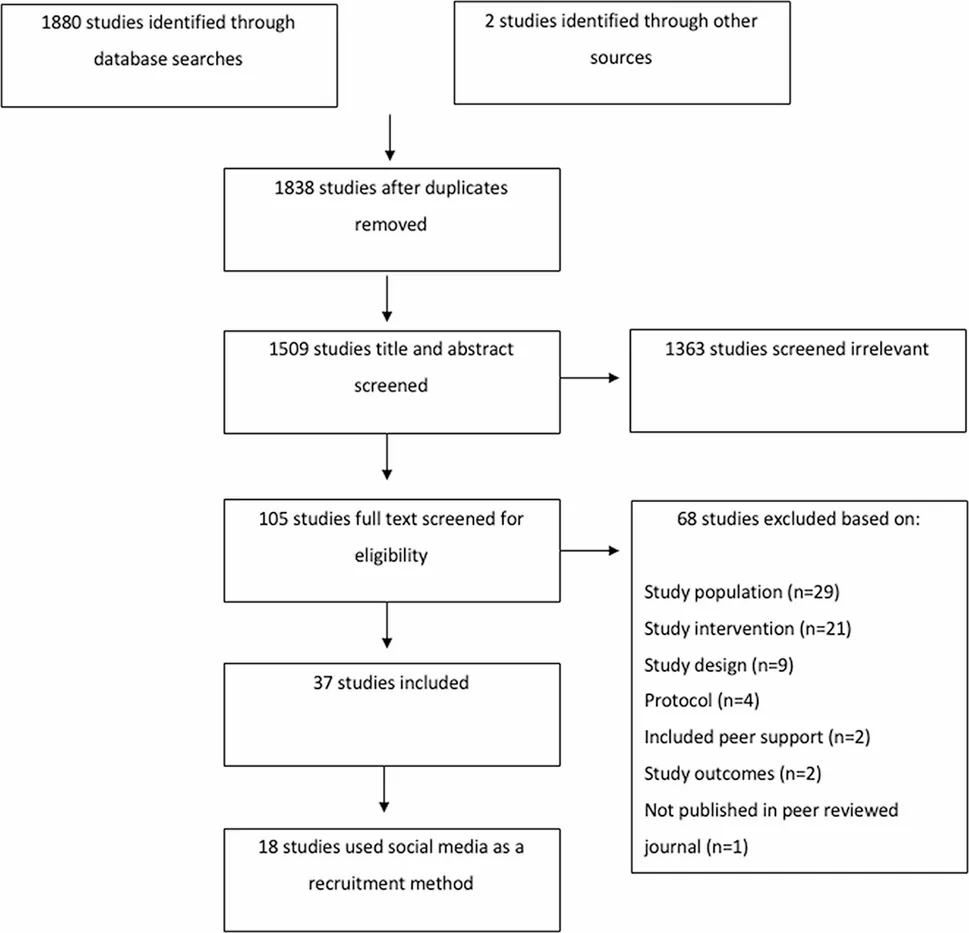

The correct Fig. 1 is.

**Figure:**
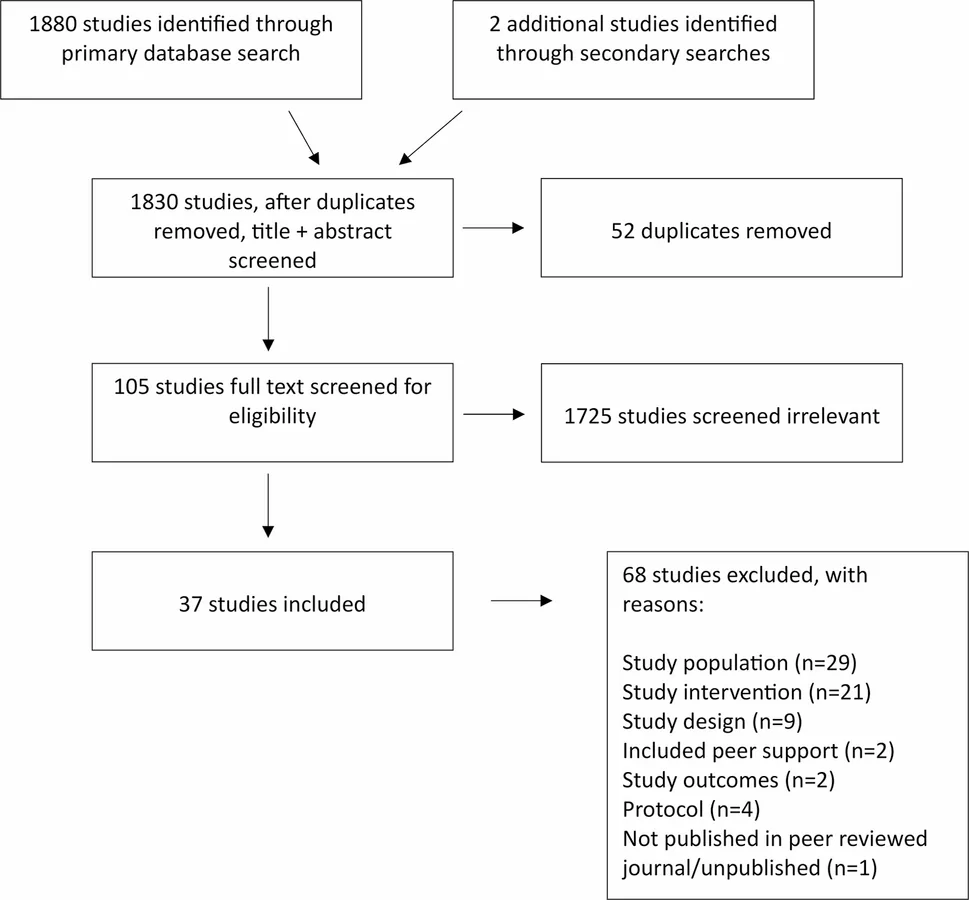

The original article[1] has been updated.
