# Supplementary material for: How have contemporary research studies used social media to recruit to digital self-help intervention research for young people’s mental health?: a mapping review
Source: BMC Med Res Methodol. 2025 Aug 29;25:203. doi: 10.1186/s12874-025-02636-9 (PMC12395651; doi:10.1186/s12874-025-02636-9)
Supplement: Supplementary file 1 — Supplementary Material 1 [file 12874_2025_2636_MOESM1_ESM.docx]

**Appendix 1**

**Table 1**

Summary of included studies, including country, study design, sample characteristics and intervention.

| Authors and Year | Country | Study Design | Sample: Participants; Age Range (Mean); Education | Intervention |
| --- | --- | --- | --- | --- |
| Abramovitch et al., 2024 | United States | Randomised Controlled Trial | 70; 18-65 (18.85); university | OCD^a^.app - anxiety, mood and sleep: An app-based 2-week perfectionism intervention designed facilitate adaptive interpretations of thoughts, emotions and events associated with anxiety, mood and sleep problems, consisting of three gamified ‘levels’ a day. |
| Bevan Jones et al., 2020 | Wales | Feasibility/Acceptability Study (including pre-post measures) | 36; 13-23 (16.3); school | MoodHwb: A multiplatform intervention designed to support young people with depression through psychoeducation, and self-management activities, with a section for parents and carers. Included illustrations, animations and interactive components. |
| Carmona et al., 2021 | Canada | Feasibility/Acceptability Study (including pre-post measures) | 51; 15-24 (20.17); university, other | DOZE^b^: A web-based app designed to address sleep disturbance among adolescents and young adults. Included a psychoeducation, a sleep diary, personalised feedback, and goal setting. |
| Chang et al., 2022 | United States | Randomised Controlled Trial | 679; ^m^–^m^ (21.3); university | A 25-minute web-based module teaching two Upa Yoga practices designed to improve student wellbeing during COVID-19. Participants were then provided with instructional videos and audio guidance and recommended to practice daily for 12 weeks. |
| Cook et al., 2019 | England | Randomised Controlled Trial | 235; 18-24 (20); university | i-RFCBT^c^: A web-based 6-module CBT^d^ intervention to address rumination by shifting thinking to become more concrete, specific and action-orientated. Included psychoeducation, mood diaries, audio exercises, and testimonials. There were two versions: guided and unguided. |
| Crosby et al., 2023 | United States | Feasibility/Acceptability Study (including pre-post measures) | 38; 18-24 (19.24); university | Sleep Scholar: A single-session, 3-module, self-guided, web-based insomnia intervention. Included sleep psychoeducation, stimulus control therapy, and sleep quality enhancement |
| Dobias et al., 2022 | United States | Randomised Controlled Trial | Project ABC = 3679, Project SAVE = 1652, The REFRAME = 848; 13-19 (Project SAVE = 15.71, other conditions = ^m^); unknown | Project ABC^e^: A 5- to 8- minute web-based single-session intervention to boost mood and build self-efficacy by encouraging engagement in pleasurable, value-aligned behaviours. Included psychoeducation, value assessment, planning and writing exercises.  Project SAVE^f^: An 8-minute web-based single-session intervention designed to reduce self-harm behaviours. Included psychoeducation, statistics and testimonials and alternative coping strategies.  The REFRAME: A 5-minute web-based single-session intervention teaching cognitive reappraisal. Included psychoeducation, testimonials, a practice exercise and prompts to apply cognitive reappraisal to their own lives. |
| Dodd et al., 2022 | United States | Randomised Controlled Trial | 167; ^m^–^m^ (19); university | CBM-I^g^ task: A 2-session intervention for perfectionism involving brief scenarios with non-perfectionistic endings. Participants attended to the non-perfectionistic endings then visualised themselves in the scenario and imagined what would happen next. |
| Finlay-Jones et al., 2023 | Australia | Randomised Control Trial | 151; 16-25 (21.15); unknown | A 4-week, self-guided, web-based self-compassion training program for improving the well-being of young people living with a chronic medical condition. Included psychoeducation, reflection and meditation exercises. |
| Fiodorova et al., 2022 | Canada | Randomised Control Trial | 105; ^m^–^m^ (18); university | A web-based 3-week intervention aiming to improve well-being. Involved a daily 5-minute self-care reflection exercise using a mood board, stress rating and check-in questions. |
| Fitzpatrick et al., 2023 | United States | Randomised Control Trial | 357; 11–14 (12.01); school | Project SOLVE: A 30-minute, web-based, self-guided single-session intervention intended to reduce internalising symptoms by strengthening adolescents’ everyday problem solving. Included psychoeducation, vignettes and practice exercises. |
| Garbett et al., 2023 | Indonesia | Randomised Control Trial | 1847; 15-19 (16.94); unknown | Warna-Warni Waktu: A single-session, social media-based intervention designed to reduce body dissatisfaction among young women, based on change techniques such as psychoeducation, media literacy and cognitive dissonance. Included videos telling a story of a woman navigating appearance pressures, with short interactive activities. |
| Harith et al., 2024 | Malaysia | Quasi Experimental | 464; 18-25 (20.88); university | EduMind: A 7-day web-based intervention tailored to participants’ individual mental health status including psychoeducation, CBT^d^ elements, breathing exercises and mood journaling. Participants were encouraged to interact with the website for 10 minutes daily, at least five times a week. |
| He et al., 2022 | United States | Randomised Controlled Trial | 148; 17-21 (18.78); university | XiaoE: A 7-day web-based artificial intelligence chatbot-guided CBT^d^ intervention for young adults with depression. The chatbot provided daily modules on topics such as cognitive distortions, self-esteem, mindfulness, loneliness and gratitude journaling. |
| Houck et al., 2022 | United States | Feasibility/Acceptability Study (including pre-post measures) | 42; 12-14 (^m^); school | iTRAC^h^: A 4-module, web-based intervention targeting sexual risk behaviours through emotional regulation skills training. Used a gaming task to increase emotional awareness and recognition and teach strategies for emotion regulation. |
| Jaycox et al., 2019 | United States | Feasibility/Acceptability Study (including pre-post measures) | 51; ^m^–^m^ (15.02); school | LIFT^i^: A 7-module web-based CBT^d^ intervention for either stress or trauma (depending on the adolescents self-reported trauma symptoms at the start of the programme). Included interactive elements, gamified tasks, goal setting and mental health assessments. |
| Kahlon et al., 2023 | Norway | Randomised controlled trial | 100; 13-16 (14.2); school | VRET^j^: A self-guided 3-week virtual reality intervention for public speaking anxiety in adolescents. Involved speech exercises with virtual audience and gamified elements. |
| Kulikov et al., 2023 | United States | Randomised Control Feasibility Trial | 51; 13-21 (intervention = 17.9; control = 16.96); school, university | Spark v2.0: A 5-week app-based BA^k^ intervention for depression in adolescents. Included psychoeducation on BA^k^, tracking features, skill building, identifying barriers and planning features. |
| Maciejewski et al., 2023 | Poland | Randomised Control Trial | 372; 18-47 (20.98); unknown | Stressbot: A self-guided 7-day web-based intervention aiming to reinforce coping self-efficacy, reduce stress and improve quality of life. Involved 6 daily exercises delivered as messages which users could reply to via text, voice or buttons. |
| March et al., 2021 | Australia | Open trial | 10366; 7-17 (12.47); school | BRAVE Self-Help: A 10-session web-based self-guided CBT^d^ program for anxiety in young people. Included psychoeducation and activities such as relaxation training, cognitive restructuring, and graded exposure. |
| Miller et al., 2023 | United States | Feasibility/Acceptability Study (including pre-post measures) | 30; 12-21 (17.03); unknown | Spark v1.0: A 5-week, self-guided BA^k^-based intervention for depression in adolescents. Psychoeducation and tasks on BA, mood tracking, mindfulness, activity scheduling problem-solving and relapse prevention were delivered through a mobile app, with corresponding virtual reality experiences through a headset. |
| Na et al., 2022 | Korea | Feasibility/Acceptability Study (including pre-post measures) | 35; 18-25 (21.66); university | MEndorphins: An 7-day app-based BA^k^ intervention for stress self-management.  Included monitoring current mood, planning stress management strategies, self-evaluation and sharing. |
| Osborn et al., 2020 | Kenya | Randomised Controlled Trial | 103; 13-18 (–^m^); school | Shamiri-Digital: a web-based self-guided single-session intervention for depression, anxiety and mental wellbeing, consisting of three modules: growth mindset, gratitude, and value affirmation. Each module included psychoeducation and a related task. |
| Papini et al., 2022 | United States | Randomised Controlled Trial | 159; ^m^–^m^ (19.27); university | AIM^l^: A two-week CBT^d^-based anxiety psychoeducation intervention including interactive components and comprehension assessments. |
| Peake et al., 2024 | United States | Randomised Controlled Trial | 160; 13-21 (intervention = 16.89, control = 16.79); unknown | Spark v2.1 and 2.2: A 5-week, self-guided BA^k^-based intervention for depression in adolescents. Provided psychoeducational content and delivered BA^k^ by teaching mood activity logging and activity scheduling. |
| Perumbil Pathrose et al., 2022 | Australia | Feasibility/Acceptability Study (including pre-post measures) | 31; 14-29 (21.65); school, university | Mindfulness-based e-book: A web-based 6-module mindfulness intervention focusing on paying attention to the five senses and self-care. The modules incorporated audios, reflection activities, and interactive quizzes. |
| Radomski et al., 2020 | Canada | Randomised Controlled Trial | 536; 13-19 (16.6); unknown | The Breathe program: A six-session web-based CBT^d^ intervention for anxiety, which included content such as relaxation, avoidance, cognitive distortions and realistic thinking. Included risk management, psychoeducation, reflection and activities. |
| Schleider et al., 2020 | United States | Feasibility/Acceptability Study (including pre-post measures) | 539; 11-17 (–^m^); unknown | Project Personality: A self-guided, 30-minute single-session intervention teaching how and why to adopt a growth mindset.  Project CARE: A self-guided, 30-minute single-session intervention teaching how and why to increase self-compassion to reduce self-hate.  The ABC^e^ Project: A self-guided, 30-minute single-session BA^k^ intervention for depression teaching how and why we should engage in valued activities. |
| Serlachius et al., 2021 | New Zealand | Feasibility/Acceptability Study (including pre-post measures) | 41; 16-30 (21.25); school, university | Whitu: An app-based 2-week CBT^d^- and PP^n^-based intervention for young people’s emotional well-being. Content included relaxation, self-compassion, gratitude and goal setting. |
| Smith et al., 2023 | Unites States | Feasibility/Acceptability Study (including pre-post measures) | 74; 13-17 (–^m^); unknown | Project Body Neutrality; A web-based, self-guided single-session intervention that aims to increase body functionality appreciation to reduce body dissatisfaction in young people. Included psychoeducation, self-reflection exercises and writing prompts. |
| Subotic-Kerry et al., 2023 | Australia | Quasi Experimental | 336; 12-15 (13.08); school | The Bite Back Mental Fitness Challenge: A 7-module, web-based, self-guided PP^n^ intervention aiming to encourage self-reflection and personal growth. Included  animations, questionnaires and activities. |
| Suffoletto et al., 2021 | United States | Randomised Controlled Trial | 52; 18-^m^ (18.7); university | MoST-MH^o^: A 3-month text message- and web-based intervention aimed to improve mental health using PP^n^, CBT^d^ and DBT^p^ strategies. Involved increasing awareness of current symptoms, stressors and their impact, and boosting self-efficacy. |
| Theurel et al., 2022 | France | Non-randomised Controlled Trial | 103; 18-25 (intervention = 20.9, control = 19.8); university | ETUCARE; an 8-week, web-based intervention designed to improve university students’ mental health during COVID-19 using strategies from CBT^d^, PP^n^, mindfulness and lifestyle medicine. Included psychoeducation and activities on topics such as stress management, procrastination, sleep and emotion regulation. |
| Torok et al., 2022 | Australia | Randomised Controlled Trial | 455; 18-25 (21.5); unknown | Lifebuoy: a 7-module, app-based self-guided DBT^p^ intervention designed to improve emotional regulation and increase distress tolerance skills. Included psychoeducation, interactive exercises and mindfulness, breathing and self-soothing tools. |
| Watkins et al, 2024 | United Kingdom, Germany, Spain and Belgium | Randomised Controlled Trial | 2532; 16-22 (19.2); school, university | An app-based intervention for mental wellbeing. There were two versions: one based on CBT^d^ principles and strategies, and one focused on emotional competence. They both included psychoeducation, self-monitoring, strategies, practise exercises, and gamified aspects. |
| Wright et al., 2019 | United Kingdom | Randomised Controlled Trial | 139; 12-18 (intervention = 14.9, control = 15.1); unknown | Stressbusters: An 8-session computer-based CBT^d^ program for depression. Each session is an interactive presentation featuring videos, animations, graphics, printouts and homework tasks. |
| Zelihifá et al, 2023 | Norway | Randomised Controlled Trial | 71; 11-18 (13.98); school | YPF^q^: A 7-session self-guided web-based intervention designed to increase psychosocial adjustment to having an appearance-affecting condition. Included advice, guidance and strategies for managing common challenges related to having a visible difference. |

^a^OCD: obsessive compulsive disorder

^b^DOZE: Delivering Online Zzz’s with Empirical Support

^c^i-RCBT: internet-based Rumination-focused Cognitive Behavioural Therapy

^d^CBT: cognitive behavioural therapy

^e^ABC: Action Brings Change

^f^SAVE: Stop Adolescent Violence Everywhere

^g^CBM-I: Cognitive Bias Modification Interpretation Retraining

^h^i-TRAC: internet-based Talking About Risk and Adolescent Choices

^i^LIFT: Life Improvement for Teens

^j^VRET: Virtual Reality exposure therapy

^k^BA: behavioural activation

^l^AIM: Anxiety Insight Modules

^m^missing data

^n^PP: positive psychology

^o^MoST-MH: Mobile Support Tool for Mental Health

^p^DBT: dialectical behavioural therapy

^q^YPF: Young Persons Face

**Appendix 2: Search terms for each database**

**PubMed**

1. Keywords and search
2. MESH headings (of keywords), select headings/ select all and search

Filter applied: years since 2019

***Search terms for PubMed***

| Column Terms  Combined with | Adolescents  AND | Common mental health/wellbeing  AND | Digital  AND | Self-help |
| --- | --- | --- | --- | --- |
| OR | Adolescent [Title/Abstract] OR  Adolescence [Title/Abstract] OR  Adolescents [Title/Abstract] | Anxiety [Title/Abstract] | E-health [Title/Abstract]  Ehealth [Title/Abstract]  E health [Title/Abstract] | Self help [Title/Abstract]  Self-help [Title/Abstract]  Selfhelp [Title/Abstract] |
| OR | Teen [Title/Abstract] OR  Teenager [Title/Abstract] OR  Teens [Title/Abstract] | Anxiety [mh] | Telemedicine [mh] | Self-help groups [mh] |
| OR | Youth [Title/Abstract]  Youths [Title/Abstract]  Youthful [Title/Abstract] | Anxiety Disorders [mh] | Computer Intervention [Title/Abstract]  Computer interventions [Title/Abstract] | Self-management [mh] |
| OR | Minor [Title/Abstract]  Minors [Title/Abstract] | Anxiety Disorder [Title/Abstract]  Anxiety disorders [Title/Abstract] | Computer assisted instruction [mh] | Self-care [mh] |
| OR | Child [Title/Abstract]  Children [Title/Abstract] | Stress [Title/Abstract] | Therapy, Computer-Assisted [mh] | Self-management [Title/Abstract]  Self management [Title/Abstract]  Selfmanagement [Title/Abstract] |
| OR | Young person [Title/Abstract]  Young people [Title/Abstract]  Young persons [Title/Abstract] | Stress Disorders [mh] | Health promotion [mh] | Self-care [Title/Abstract]  Self care [Title/Abstract]  Selfcare [Title/Abstract] |
| OR | Adolescent [mh] | Stress, psychological [mh] | Internet intervention [Title/Abstract]  Internet interventions [Title/Abstract] | Guided [Title/Abstract] |
| OR | Child [mh] | Stress, Physiological [mh] | Internet | Guided self-help  [Title/Abstract] |
| OR | College student  [Title/Abstract]  OR  College student  [Title/Abstract] | Affective Disorders [Title/Abstract] | Digital health intervention [Title/Abstract]  Digital health interventions [Title/Abstract] | Modules  [Title/Abstract] |
| OR | University student  [Title/Abstract]  OR  University students  [Title/Abstract] | Mood Disorders [mh] | Online intervention [Title/Abstract]  Online interventions [Title/Abstract] | Self help techniques  [Title/Abstract]  OR  Self-help techniques  [Title/Abstract] |
| OR | Young adult  [Title/Abstract]  OR  Young adults  [Title/Abstract] | Mental Disorders [mh] | Mobile application [Title/Abstract]  Mobile applications [Title/Abstract] |  |
| OR | Student  [Title/Abstract]  OR  Students  [Title/Abstract] | Mood [Title/Abstract] | Medical Informatics Applications [mh] |  |
| OR | Student (mh) | Affect [mh] | Tablet intervention [Title/Abstract]  Tablet interventions [Title/Abstract] |  |
| OR |  | Affective symptoms [mh] | Electronic Health [Title/Abstract]  Electronic-health [Title/Abstract] |  |
| OR | Young adult (mh) | Depression [Title/Abstract] | Digital intervention [Title/Abstract]  Digital interventions [Title/Abstract] |  |
| OR |  | Depression [mh] | Computer Systems [mh] |  |
| OR |  | Wellbeing [Title/Abstract]  Well-being [Title/Abstract]  Well being [Title/Abstract] | Telephone [mh] |  |
| OR |  | Distress [Title/Abstract] | Wireless Technology [mh] |  |
| OR |  | Mental health  [Title/Abstract] | Electronic intervention [Title/Abstract]  Electronic interventions [Title/Abstract] |  |
|  |  | Mental health {mh) | Online  [Title/Abstract] |  |
| OR |  | Psychopathology  [Title/Abstract] | Digital  [Title/Abstract] |  |
| OR |  | Psychopathology (mh) | Computerised  [Title/Abstract] |  |
| OR |  | Mental wellness  [Title/Abstract] | Technology-based intervention  [Title/Abstract]  OR  Technology based intervention  [Title/Abstract] |  |
| OR |  | Mental illness  [Title/Abstract] | Telehealth  [Title/Abstract] |  |
| OR |  | Mental disorder  [Title/Abstract] | mHealth  [Title/Abstract] |  |
| OR |  | Internalising  [Title/Abstract]  OR  Internalizing  [Title/Abstract]  OR  Internalising disorder  OR  Internalizing disorder | eHealth  [Title/Abstract] |  |
| OR |  | Psychiatric disorder  [Title/Abstract] | Web-based  [Title/Abstract]  OR  Web based  [Title/Abstract] |  |
| OR |  | Psychiatric illness  [Title/Abstract] | Internet-based  [Title/Abstract]  Internet based  [Title/Abstract] |  |
| OR |  | Reduced wellbeing [Title/Abstract] | Mobile phone  [Title/Abstract] |  |
| OR |  | Disorder  [Title/Abstract]  OR  Disorders  [Title/Abstract] | Text message  [Title/Abstract]  OR  Text messaging  [Title/Abstract] |  |
| OR |  | Worry  [Title/Abstract] | Text messaging (mh) |  |
| OR |  |  | SMS  [Title/Abstract] |  |
| OR |  |  | Cell phone  [Title/Abstract]  OR  Cellular phone  [Title/Abstract] |  |
| OR |  |  | Cell phone (mh) |  |
|  |  | **AND** | **AND** | **AND** |

**APA PsycInfo**

1. TITLES & ABSTRACT search
2. MESH headings (of keywords), select headings/ select all and search

Filter applied: years since 2019

***Search terms for APA PsychInfo***

| Column Terms  Combined with | Adolescents  AND | Common mental health problems/wellbeing AND | Digital  AND | Self help |
| --- | --- | --- | --- | --- |
| OR | Adolescen* | Anxiety | E?health | Self?help |
| OR | Teen* | Anxiety (MeSH) | Telemedicine (MeSH) | Self-help groups (MeSH) |
| OR | Youth* | Anxiety Disorders (MeSH) | Computer Intervention* | Self-management (MESH) |
| OR | Minor* | Anxiety Disorder* | Computer assisted instruction (MESH) | Self-care (MeSH) |
| OR | Child* | Stress | Therapy, Computer-Assisted (MESH) | Self?management |
| OR | Young pe* | Stress Disorders (MESH) | Health promotion (MeSH) | Self?care |
| OR | Adolescent (MeSH) | Stress, psychological (MeSH) | Internet intervention* | Guided |
| OR | Child (MeSH) | Stress, Physiological (MESH) | Internet | Guided self?help |
| OR |  | Affective Disorders | Digital health intervention* | modules |
| OR | College student | Mood Disorders (MeSH) |  | Self-help techniques |
| OR | University student | Mental Disorders (MESH) | Online intervention* |  |
| OR | Young adult | Mood |  |  |
| OR | Student | Affect (MESH) | Mobile application* |  |
| OR |  | Affective symptoms (MESH) | Medical Informatics Applications (MeSH) |  |
| OR |  | Depression | Tablet intervention* |  |
| OR |  | Depression (MESH) | Electronic?Health |  |
| OR |  | Well?being | Digital intervention* |  |
| OR |  | Distress | Computer Systems (MeSH) |  |
| OR |  | Mental health | Telephone (MeSH) |  |
| OR |  | Psychopathology | Wireless Technology (MeSH) |  |
| OR |  | Mental wellness | Electronic intervention* |  |
| OR |  | Mental illness | Online |  |
| OR |  | Mental disorder | Digital |  |
| OR |  | Internalising | Computerised |  |
| OR |  | Internalizing | E?therapy |  |
| OR |  | Reduced wellbeing | Technology based intervention |  |
| OR |  | Worry | Telehealth |  |
| OR |  |  | mHealth |  |
| OR |  |  | eHealth |  |
| OR |  |  | Web?based |  |
| OR |  |  | Internet?based |  |
| OR |  |  | Mobile phone |  |
| OR |  |  | Text message |  |
| OR |  |  | SMS |  |
| OR |  |  | Cell phone |  |
|  |  | **AND** | **AND** | **AND** |
